# Can Foraminal Stenosis Due to Lumbar Isthmic Spondylolisthesis Cause Axonopathy in the Lower Extremity?

**DOI:** 10.3390/healthcare9050511

**Published:** 2021-04-28

**Authors:** Seong Hyeon Jo, Jang Hyuk Cho, Dong Gyu Lee

**Affiliations:** 1 Department of Physical Medicine and Rehabilitation, College of Medicine, Yeungnam University, Daegu 42415, Korea; cshyun123@naver.com; 2 Department of Rehabilitation Medicine, Keimyung University School of Medicine, Keimyung University Dongsan Hospital, Daegu 42601, Korea; chojang75@hanmail.net

**Keywords:** lumbar spine, foraminal stenosis, electrodiagnostic study, nerve conduction study, axonopathy

## Abstract

This study aimed to investigate, using electrodiagnosis, whether foraminal stenosis due to isthmic spondylolisthesis (IS) causes peripheral nerve axonopathy. We retrospectively reviewed the medical records of the Yeungnam University Hospital and included 46 patients (mean age = 60.8 ± 13.7 years; male:female = 24:22) with foraminal stenosis due to IS. We classified foraminal stenosis grading based on T2 and T1 sagittal spinal magnetic resonance imaging (MRI). Patients were divided into mild (n = 18) and severe foraminal stenosis (n = 28) groups. To evaluate axonopathy in the lower extremity, results of compound motor action potential (CMAP) of the extensor digitorum brevis muscle (EDB) and abductor hallucis brevis muscle (AHB), and sensory nerve action potential (SNAP) of the sural nerve were retrieved. No statistically significant difference was observed in the amplitude of CMAP of the EDB and AHB and SNAP of the sural nerve with the severity of foraminal stenosis. However, age showed a statistically significant relationship with the amplitude of NCS in the EDB, AHB, and sural nerves (*p* < 0.001). The severity of foraminal stenosis due to IS showed no relationship with axonopathy beyond age-related degeneration of the lower extremities. Therefore, if there is robust axonopathy in lower extremities, physicians should consider pathologies other than foraminal stenosis due to IS.

## 1. Introduction

Spondylolisthesis is the forward displacement of the vertebra adjacent to it. According to the type of spondylolisthesis, lesions of spinal stenosis can be central, subarticular, and foraminal [1]. Peripheral entrapment neuropathy arises from compression along the nerve pathway. Localized external compression forces cause mechanical deformation and ischemia, resulting in demyelination and axonal injury [2]. Spinal stenosis and spondylolisthesis show anatomical features similar to those of peripheral entrapment neuropathy.

Chronic nerve compression produces pain and loss of function in the distal parts of the compressed nerves. Chronic nerve compression injury induces demyelination and apoptosis in the lesions caused by the injury. Subsequently, concurrent Schwann cell reactivation results in remyelination and provokes nerve recovery. Nerve injury following entrapment causes pain and functional deterioration of the innervated structures. Spinal stenosis can cause claudication or radicular pain. This conceptual similarity between peripheral nerve entrapment syndrome and spinal stenosis suggests that foraminal stenosis causes axonal injury in the spinal nerve.

There are some morphological and vascular differences between the peripheral and central nervous systems. The spinal cord and nerve roots are covered by three meninges: the dura mater, arachnoid mater, and pia mater. Although peripheral nerves also have three layers of sheath, blood–brain barriers showed more robust chemical defense in the spinal nerve root than the peripheral nerve sheath [3]. Moreover, the spinal nerve root had less endoneurial collagen, less arteriolar and venular networks, and a hypovascularity zone compared to the peripheral nerve [4]. Clipping of spinal nerve at midpoint between the dural sac and dorsal root ganglion provoked central chromatolysis and functional deterioration of dorsal root ganglion [5]. The DRG and spinal root are located within the spinal foramen. Therefore, we speculated that severe spinal foraminal stenosis could cause axonal injury of sensory nerve.

Some researchers have reported that spinal stenosis or disc pathology produces axonopathy or paraspinal muscle denervation of the peripheral nerves of the affected extremities [6]. Based on this notion, spinal stenosis can cause axonal injury. However, despite an existing anatomical deformity compressing the spinal nerve, spinal stenosis does not always produce motor and sensory symptoms. Moreover, in previous research, central stenosis did not show a relationship with peripheral neuropathy in the lower extremities [7]. Therefore, it is controversial whether spinal stenosis affects spinal root injury, subsequently resulting in peripheral nerve axonopathy.

The differential diagnosis of spinal pathologies is usually challenging. Nerve conduction studies are used to diagnose peripheral nerve pathologies for the differential diagnosis of spinal pathologies as supplementary and auxiliary tools [8]. Therefore, a reasonable and accurate interpretation of the findings of electrodiagnosis is needed for physicians to make a differential diagnosis.

Degenerative spondylolisthesis (DS) causes central, subarticular, and/or foraminal stenosis. However, isthmic spondylolisthesis (IS) often leads to foraminal stenosis in the slipped area. The central and peripheral nerves have different sheath structures. The meninges and perineurium cover the central and peripheral nervous systems, respectively. The dorsal root ganglion (DRG) is located and passes through the spinal foramen [9]. DRG is the transitional area of the neural sheath from the meninges to the perineurium [10]. Based on these anatomical differences, if foraminal stenosis causes axonal injury of the spinal nerve, the nerve corresponding to the level of foraminal stenosis shows abnormal findings in the amplitude in the motor and sensory nerve conduction study (NCS). Therefore, we investigated whether foraminal stenosis caused by IS causes axonopathy of the peripheral nerve using electrodiagnosis.

## 2. Materials and Methods

The subjects were selected from medical records obtained from Yeungnam University Hospital from January 2016 to November 2020. Ethical approval for the study was obtained from the hospital’s institutional review board and the inclusion criteria were as follows: 1. diagnosed spondylolisthesis at the L5, 2. Having undergone spinal MRI evaluation, and 3. Having undergone an electrodiagnostic exam. The exclusion criteria were as follows: 1. history of peripheral neuropathy (peripheral entrapment syndrome, acute inflammatory demyelinating polyneuropathy, metabolic peripheral axonopathy, chemotherapy); 2. degenerative spondylolisthesis, 3. disc herniation, 4. spine fracture, 5. cauda equina syndrome; 6. diabetes; 7. history of spinal surgery. Among the 1941 patients, 46 met the inclusion and exclusion criteria (Table 1).

### 2.1. Assessments

Four grades of foraminal stenosis (grade 0, normal; 1, a mild degree of foraminal stenosis; 2, a moderate degree of foraminal stenosis; 3, a severe degree of foraminal stenosis) were evaluated on the findings of sagittal MRI [11]. The MRI foraminal stenosis grading system was based on the fat obliteration of foraminal area and morphologic change of the spinal nerve. If there was a morphological change in the spinal nerve without perineural fat obliteration in four directions, we assigned it as grade 3 foraminal stenosis. Two physicians agreed and evaluated the foraminal stenosis grading at the spinal level of IS. We divided the patients into two groups based on the severity of the foraminal stenosis. The mild group was classified as grade 0–2 and the severe groups were classified as grade 3 (Table 1). As in peripheral nerve entrapment, the change of nerve morphology is reported to be related with axonopathy [12,13].

NCS (Carefusion Nicolet EDX with Viking EDX software, Middleton, WI, USA) on the lower extremities was conducted on all patients. Compound motor action potential (CMAP) and sensory nerve action potential (SNAP) were used to assess peripheral axonopathy caused by foraminal stenosis.

### 2.2. Statistics

Statistical analysis was performed using JAMOVI version 1.6 [14]. The continuous variables were described as means ± standard deviations (SDs). The independent *t*-test and Mann–Whitney U test were used to evaluate whether the two groups (mild or severe) had different distributions of age and NCS. Pearson’s s correlation analysis was used to assess the relationship among age, the severity of foraminal stenosis, and the amplitude on the NCS. The foraminal stenosis group was dichotomous; thus, a Point-Biserial correlation analysis was used to analyze the relationship between the foraminal stenosis groups and other variables. The results were considered statistically significant if *p*-values were less than 0.05.

## 3. Result

A total of 46 participants were included in this study (Table 1). The mean age was 60.8 ± 13.7 years. Based on the MRI grading system, the distribution of foraminal stenosis grading was as follows: level 0: 7 (15.2%), level 1: 4 (8.7%), level 2: 7 (15.2%), and level 3: 28 (60.9%). There were 18 and 28 patients in the mild and severe groups, respectively.

**Figure 1:**
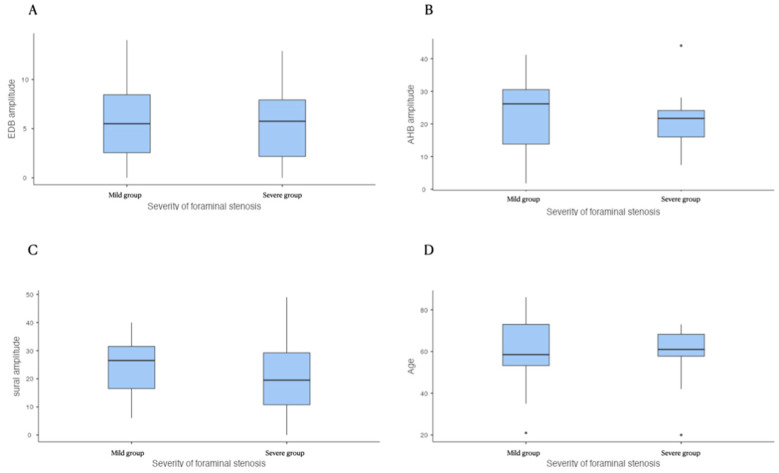
Between the two groups of foraminal stenosis, there is no significant difference in the EDB (**A**), AHB (**B**), and sural amplitude (**C**) and age (**D**). EDB: extensor digitorum brevis, AHB: abductor hallucis brevis.

**Figure 2:**
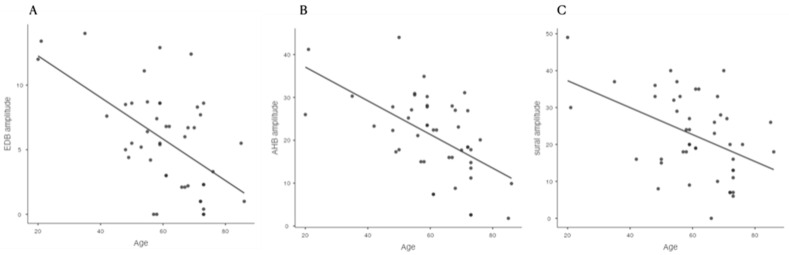
Age showed a statistically significant correlation with the amplitude of the EDB (**A**), AHB, (**B**) and sural nerve (**C**). EDB: extensor digitorum brevis, AHB: abductor hallucis brevis.

The severity of foraminal stenosis did not show a statistically significant difference with the amplitude of CMAP of the EDB and AHB and SNAP of the sural nerve (Table 2 and Figure 1). Each group did not show a statistically significant age difference. However, age showed a statistically significant relationship with the amplitude of NCS on the EDB, AHB, and sural nerve (*p* < 0.001, Table 3). There was a negative correlation between age and the NCS amplitude (Figure 2). These results indicate that age is the key factor influencing the NCS amplitude not the morphologic change of spinal nerve. However, foraminal stenosis group did not show relationship with amplitude of NCS on lower extremity.

## 4. Discussion

In our study, age was negatively correlated with the amplitude in the NCS on the motor and sensory nerves. However, foraminal stenosis did not have a relationship with the amplitude in the NCS on the nerves of the lower extremities. The foraminal stenosis group did not show a statistically significant age difference. Therefore, the severity of foraminal stenosis caused by IS did not have the relationship with the amplitude of nerves on lower extremities.

Several studies have reported that spinal stenosis shows abnormal findings upon electrodiagnostic examinations (EDX). Among EDX, needle electromyography showed clinically significant findings with spinal stenosis [15]. Sensory evoked potential (SEP) or H reflex showed abnormal findings in patients with spinal stenosis [16]. However, abnormal findings of needle electromyography can be improved over time in patients with disc herniation [17]. Moroever, EDX may be used to show non-specific findings at the beginning of the clinical manifestation of spinal stenosis. Therefore, concurrent pathological examination with spinal stenosis leads to inflammation or acute nerve injury and can provoke abnormal findings on needle electromyography. Even in studies reporting the usefulness of EDX in spinal stenosis, subjects with spinal stenosis did not show a statistically significant reduction in the CMAP or SNAP compared with normal subjects [18]. In our study, the mild groups did not show statistically significant differences compared to the severe group. Moreover, the amplitude of the NCS did not correlate with the severity of foraminal stenosis but rather with age. Therefore, it has been challenging to diagnose spinal stenosis using the EDX [19].

The severity and shape of spinal stenosis can improve with a change in posture [20]. Moreover, gait or standing can provoke neurogenic claudication. The nerve conduction time was reduced in spinal stenosis patients after exercise, which proves that spinal stenosis dynamically affects the cauda equina and/or spinal nerve [21]. However, pre-exercise and motor-evoked potential latency time did not differ between stenosis patients and healthy volunteers. Even in patients with spinal stenosis on imaging studies and symptoms, the spinal nerves are affected depending on the patient’s posture or situation. As EDX are affected by dynamic movements of the spine, there can be limitations in evaluating NCS findings based on the degree of stenosis observed by static imaging.

It is well known that aging affects the functional ability and morphology of the peripheral nervous system [22]. Aging causes nerve fibers to shrink, reduces nerve diameter, and alters the fiber membrane. The length of the peripheral nerves accelerates length-dependent degenerative neuropathy. Therefore, aging itself is an independent factor for peripheral neuropathy in the lower extremities [23,24,25]. We wanted to know whether spinal foraminal stenosis influences peripheral nerve axonopathy beyond age-related peripheral nerve degeneration. Aging causes a decline in the nerve regeneration power following nerve injury. Therefore, we speculate that the lower recovery rate of spinal nerve injury caused by foraminal stenosis affects the result of NCS in aging patients.

The aging population has higher incidence of spinal stenosis and ageing is the one of factors for the chronic pain [26,27]. Therefore, the elderly have a higher incidence of decompressive operation for radicular and axial back pain. Considering that there is a symptom-free population despite stenosis images, physician should be careful in their diagnosis and strategy for pain treatment for spinal stenosis. EDX has been conducted for the differential diagnosis to the radicular pain [28]. Precise interpretation of EDX increases the quality and accuracy of the diagnosis and treatment. Although trauma, disc herniation, or acute aggravation of radicular symptoms can produce abnormal findings on EDX, this study showed that ageing itself has higher relationship with the NCS than the severity of foraminal stenosis.

Our study has some limitations. First, the subjects were retrospectively recruited. Therefore, we cannot exclude patients who had a factors influencing peripheral nerve injury like smoking, or occupational history causing peripheral nerve damage. Second, functional evaluation and spinal instability were not considered. Spinal MRI was conducted at a static position of the spine. The spine has dynamic motion so that the severity of spinal stenosis can differ between daily activity conditions and static MRI image findings. Therefore, it is challenging to accurately evaluate the severity of foraminal stenosis.

In conclusion, the severity of foraminal stenosis due to IS did not show a relationship with axonopathy beyond the age-related degeneration of the lower extremities. Therefore, if there is robust axonopathy in the lower extremities, physicians should consider pathologies other than spinal stenosis.

## Figures and Tables

**Table 1:** Demographic data.

		Total Patients	Foraminal Stenosis Grade	
			Mild Group	Severe Group
Patients		46	18	28
Age		60.8 ± 13.7	61.0 ± 16.9	60.6 ± 11.4
Sex (M:F)		24:22	9:9	15:13
Foraminal stenosis grade	0	7 (15.2%)	7	
	1	4(8.7%)	4	
	2	7(15.2%)	7	
	3	28(60.9%)	0	28

IS: isthmic spondylolisthesis.

**Table 2:** The amplitude in the NCS on lower and age according to the severity of foraminal stenosis.

Variables	Foraminal Stenosis		
	Mild (18)	Severe (28)	
	Mean ± SD	Mean ± SD	*p*
CMAP EDB (mV)	6.02 ± 4.19	5.50 ± 3.73	0.662
CMAP AHB (mV)	21.8 ± 12.0	20.6 ± 7.65	0.672
SNAP sural nerve (μV)	24.8 ± 9.81	20.9 ± 11.7	0.245
Age	61.0 ± 16.9	60.6 ± 11.4	0.839

CMAP: compound motor action potential, EDB: extensor digitorum brevis, AHB: abductor hallucis brevis, SNAP: sensory nerve action potential, NCS: nerve conduction study.

**Table 3:** Correlation matrix among age, severity of foraminal stenosis, and amplitude in the peripheral nerve conduction study.

Variables		Age		EDB Amplitude		AHB Amplitude		Sural Amplitude
Age	r	-						
	*p*-value	-						
EDB amplitude	r	−0.566	*	-				
	*p*-value	<0.001		-				
AHB amplitude	r	−0.568	*	0.648	*	-		
	*p*-value	<0.001		<0.001		-		
sural amplitude	r	−0.451	*	0.595	*	0.420	*	-
	*p*-value	0.002		<0.001		0.004		-
Foraminal stenosis group	r	−0.013		−0.066		−0.064		−0.175
	*p*-value	0.932		0.62		0.670		0.245

CMAP: compound motor action potential, EDB: extensor digitorum brevis, AHB: abductor hallucis brevis, SNAP: sensory nerve action potential. r, correlation coefficient. The result table of the foraminal stenosis group shows a Point-Biserial correlation. * *p* < 0.05.

## Data Availability

The datesets used and/or analyzed during the current study are available from the corresponding author on reasonable request.
